# Various hypotheses on MHC evolution suggested by the concerted evolution of CD94L and MHC class Ia molecules

**DOI:** 10.1186/1745-6150-1-3

**Published:** 2006-01-31

**Authors:** Etienne Joly

**Affiliations:** 1 Equipe de Neuro-Immuno-Génétique Moléculaire, IPBS, UMR CNRS 5089, 205 route de Narbonne, 31077 Toulouse Cedex, France

## Abstract

**Background:**

In the accompanying paper by Virginie Rouillon and myself, our demonstration that homogenisation by gene conversion occurs readily among MHC class I genes was made possible because of the exceptional conservation of the CD94L locus between divergent species of separate taxa, suggesting that the molecules of this family are endowed with very important and well preserved biological functions. These results lead me to elaborate various hypotheses on several aspects of MHC evolution.

**Hypotheses:**

In a first part, I propose a highly hypothetical scenario of MHC evolution that could explain how modern day CD94L molecules can have so many diverse and well preserved biological functions. Next, I propose that MHC class I molecules evolve more rapidly and exuberantly than class II molecules because the former are subjected to more direct selective pressures, in particular from viruses. Third, I suggest that concerted evolution, by increasing inter-genic homogeneity would in turn favour further inter-allelic and inter-loci exchanges, hence resulting in a more evolvable MHC. As a fourth and last point, I propose that the high GC content of sequences coding for classical class I molecules could be a consequence of biased gene conversion.

**Testing of these various hypotheses **should occur naturally over the coming years, with the ever increasing availability of more sequences related to MHC class I genes from various organisms. Ultimately, a better understanding of how MHC molecules evolve may help to decipher where and how our adaptive immune system arose, and keeps evolving in the face of the permanent challenge of infectious organisms.

**Reviewers:**

This article was reviewed by Stephan Beck, Lutz Walter and Pierre Pontarotti.

## Open peer review

Reviewed by Stephan Beck, Lutz Walter and Pierre Pontarotti.

For the full reviews, please go to the Reviewers' comments section.

## What place do modern day CD94L molecules occupy in the history of MHC evolution?

HLA-E and/or H-2Qa1 fulfil many other roles besides that of presenting the leader peptides of class Ia molecules to NK cells. They present leader peptides derived from Hsp60 heat shock proteins of self or bacterial origin [1] to cytotoxic T cells [2]. This presentation of leader peptides from Hsps to NK cells has also been proposed to play a role in stress surveillance [3]. HLA-E also presents peptides derived from viruses [4] or bacteria [5] to CD8+ cytotoxic T cells and to NKT cells [6], and H-2Qa1 aids the resistance of mice to *Salmonella *infection by presenting antigens to γδ T cells [7] and regulates the activity of CD8 regulatory T cells [8]. The capacity of CD94L molecules to fulfil so many functions suggests that they have probably been around for a very long time. Yet, despite the daily increase in availability of additional sequences from various species, we have failed to identify clear homologues of the modern day CD94L found in rodents and primates in animals from other orders, including in the complete dog genome. This would tend to suggest that an ancestral CD94L molecule arose in a rodent-primate ancestor that lived after the divergence of the Euarchontoglires (a clade that includes primates and rodents) from the laurasiatheria (comprising carnivores, ongulate herbivores and bats)[18].

Here, I would like to present a highly hypothetical scenario of MHC evolution that could explain how the CD94L molecules found in rodents and mammals today are endowed with so many diverse functions. This scenario is based on the recent discovery of a molecule related to CD94 in a urochordate, [9], which lends support to the view that some sort of NK cells (with their receptors) existed before adaptive lymphocytes.

Around 800 million years ago, there lived the ancestor of urochordates (sea squirts), and cephalochordates (Amphioxus, or lancelets, small eel-like sea-dwelling life forms with an unsegmented backbone). In its turn, 50 million years later, the ancestor of vertebrate fish would evolve from the ancestor of cephalochordates. In this urochordate-cephalochordate ancestor, I propose that the ancestor of MHC molecules presented hydrophobic peptides (including leader peptides of self and non-self origin) to an ancestral CD94 receptor, with a role in stress and/or danger detection. Consistent with this, Flajnik *et al. *have previously proposed that ancestral MHC molecule(s) derived from heat shock proteins (Hsp) [10], which have particularly high affinities for hydrophobic peptides. Although the proposal by Flajnik *et al. *was based on tenuous sequence homologies, and later elucidation of an Hsp crystal structure [11] revealed that Hsps and MHC molecules bind to peptides in very different fashions, this type of hypothesis remains interesting, if only because Hsps do behave as danger signals when they are released in the extracellular milieu, in line with a recent hypothesis regarding the hydrophobic nature of danger signals [12].

Vertebrates arose around 750 million years ago, in the form of jawless fish, of which lampreys and hagfishes are modern day representatives. The discovery of an invariable TCR-like molecule in sea lamprey suggests that this type of molecule had already evolved by then [13]. The ligand(s) of this prototypic TCR has yet to be identified, and it will also be particularly interesting to find out if jawless fish express CD94-like receptor molecules. For our imagined scenario, I envisage that the ancestors of MHC molecules started presenting hydrophobic peptides to a TCR-like protein and to the CD94-like invariable receptors as part of an innate immune system geared at detecting stress and/or danger.

Lampreys have recently been shown to possess an adaptive immune system [14,15]. The variable receptors expressed by lamprey lymphocytes are, however, not related to TCRs or to any other members of the immunoglobulin superfamily, but are based on somatic genomic recombinations of fragments coding for leucine-rich repeats. The appearance of a segmented spinal chord apparently correlated somehow with a much-increased need for an adaptive immune system, and the striking dichotomy between the adaptive immune system of jawless and jawed vertebrates provides a very good example of what I would call convergent evolution, i.e. the development of similar functions through two clearly independent evolutionary pathways. Another good example of convergent evolution is the acquisition of flight in bats, birds and butterflies.

Jawed vertebrates appeared around 500 million years ago and, with them, the complete adaptive immune system that can be found in all gnathostomes from mammals to cartilaginous fish (sharks). One of the more challenging current enigmas for evolutionary immunogeneticists is to understand this evolutionary leap which took place in the ancestor of jawed vertebrates and led to an adaptive immune system complete with recombination-activating genes (RAG), immunoglobulins, hypervariable TCRs, and their functional partners, the MHC class I and class II molecules [16,17]. It is interesting to note that *en bloc *genome duplications apparently occurred both between protochordates and vertebrates, and also between jawless and jawed vertebrates [16]. I envisage that, in ancestral jawed fish, as TCRs became variable because of newly evolved RAG genes, their ancestral function to detect danger signals could have quickly evolved towards the recognition of ligands more diverse than hydrophobic peptides presented by proteins related to heat shock proteins. To favour the diversification of the antigens presented, combining a hydrophobic peptide binding site to an immunoglobulin domain (the α3 domain) would have resulted in the structure found today in MHC class I molecules.

MHC molecules encoded by individual loci derived from duplication would then have become available to co-evolve with certain lymphocytes' receptors, most probably various forms of γδ TCRs. The subsequent appearance of αβ TCRs gave rise to a further diversity of receptors, matched by the appearance of MHC class Ia molecules, with two levels of diversity: degenerate binding motifs, and heterogeneity in the population.

Much later, in a rodent-primate ancestor that lived after the divergence of the Euarchontoglires, a new function arose in NK cells to ensure the maintenance of expression of MHC class Ia molecules via presentation of their leader peptides by CD94L molecules. This was achieved by virtue of a heterodimeric receptor combining the capacity of CD94 to detect hydrophobic peptides, and that of NKG2 molecules to bind certain MHC molecules expressed when cellular stress levels rise [19]. This binding function of CD94-NKG2 heterodimeric receptors to an MHC class Ib molecule presenting the class Ia leader peptides must represent a sufficient selective advantage to have been conserved over the 90 million years since the rodent/primate split. Form this, I would predict that CD94L molecules are likely to be identified in lagomorphs and lemurs since these two taxa split respectively from rodents and from other primates after the primate-rodent separation [18].

The elaborate process by which NK cells survey whether or not cells express the leader peptides of MHC class Ia molecules is quite amazing from an evolutionary point of view. The sequence of events described above explains how this could have arisen by small incremental evolutionary steps, in true Darwinian fashion [20]. In addition, this scenario would explain how modern day CD94L molecules come to have so many diverse functions, most of them related to those of ancestral MHC molecules presenting hydrophobic, danger-related peptides, from which they would be direct descendants.

## Why do MHC class I molecules evolve faster than class II ?

A general observation from comparing MHCs from different species is that the evolution of MHC class I molecules is much more rapid and exuberant than that of their class II counterparts. This is relatively easy to reconcile with the concept that MHC evolution is mostly driven by pressures imposed by infectious pathogens such as rapidly evolving viruses. Indeed, if a mutated virus acquires that capacity to evade or block MHC class I antigen presentation, the progeny of that virus will have an immediate advantage over the viruses infecting the adjacent cells. If the evasion mechanism concerns class II presentation, however, virions arising will still carry epitopes for TCR recognition by CTLs, and antigen presentation and local inflammation will still be taking place because the infection with unmutated viruses will still be happening in adjacent cells. The advantage of mutated pathogens having acquired the means to evade class II presentation will therefore only become effective if and when they reach new sites of infection, either in a separate host, or possibly at a separate site of the same host. It is therefore of no surprise that the vast majority of viral genes that can block antigen presentation target the class I pathway [21,22]. The direct consequence of this is that MHC class I molecules must be subjected to very intense selective pressures, which can in turn explain why they evolve faster than MHC class II molecules, and, for that matter, much faster than any other genes in the vertebrate kingdom.

## Concerted evolution, genomic organisation and evolvable MHCs

In our accompanying paper, we have shown that gene homogenisation, through repeated events of gene conversion, can contribute significantly to the relatedness of separate MHC class I loci within species, and we have proposed that this may favour the co-evolution of MHC class I molecules and of other protein involved in antigen presentation. Here, I would like to propose that, in a roundabout kind of way, the MHC region could have evolved to promote the evolvability of the genes within it, by favouring frequent interallelic and intergenic gene conversion. This concept of 'evolvability' as a selectable trait has recently been validated mathematically by Earl and Deem [23].

The two main factors that favour the occurrence of gene conversion between two sequences are their genomic proximity [24], and their degree of sequence homology. If we consider that the evolvability of MHC molecules could represent a selective advantage at the population level, we then see that carrying a set of genes that are prone to undergo gene conversion with one another could provide such an advantage to a given species. In most vertebrates, virtually all genes coding for MHC molecules are found within this one region defined as the MHC. The raison d'être of this situation may be to promote the occurrence of inter-genic gene conversion, thereby providing a set of genes that are particularly prone to evolve under the selective pressures imposed by pathogens.

For certain MHC molecules presenting invariable antigens, however, the tendency of the loci within the MHC to undergo frequent gene conversion with their neighbours could be disadvantageous. For example, CD1 molecules, which present invariable lipid antigens, are encoded outside the MHC in mammals, but within the MHC in chicken [25,26]. This suggests that CD1 genes may have initially been within the MHC, but that in Mammals, the CD1 loci may have 'moved out' of the MHC so as not to undergo concerted evolution with their MHC-encoded counterparts. If this scenario turns out to be true, it would bring strong support to the view that concerted evolution of sequences within the MHC contribute very actively to the shaping of MHC molecules. There are, however, at least two other possible scenarios to explain this difference found in the location of CD1 genes outside the MHC in mammals and close to it in chicken. First, as suggested by large scale analyses of whole genomic regions, the MHC and the region carrying the CD1 loci (1q21-q25) may have arisen via whole chromosomal duplications [16,27]. If this were the case, the finding of the CD1 loci within the chicken MHC suggests that those would have later been rejoined with their classical counterparts. Yet another possibility (suggested by Jim Kaufman) is that both types of loci could have existed in the ancestral MHC, but that, after duplication of the whole region, CD1 and classical MHC loci were conserved on the same chromosome in the lineage leading to chickens, but individually on the two separate chromosomes in the lineage leading to mammals. Regarding these various hypotheses, it will be interesting to find out if CD1 loci are also adjacent to MHC class I loci in other bird species, and to look for signs of concerted evolution between the MHC class I and CD1 loci in chicken and in other bird lineages.

As mentioned earlier, the occurrence of gene conversion between two DNA sequences also depends greatly on their degree of similarity. By ensuring the maintenance of a high degree of sequence homology between the various MHC loci within the region, frequent intergenic gene conversion would favour the occurrence of further conversion events, thereby conferring a selective advantage in terms of evolvability of MHC genes without needing a direct selective advantage in terms of expressed proteins. This could explain the existence and maintenance of so many pseudogenes sequences within most mammalian MHC regions, and the intra-locus homogenisation reported for introns of the HLA-A locus [28]. A rapid comparison of the HLA-E introns found them to be much more closely related to those of HLA-A than to those of H2-Qa1 and RT-BM1 (data not shown). This observation can be seen as further evidence for the extensive occurrence of intergenic homogenisation within the MHC, even outside of the coding regions.

Selection for high evolvability probably contributes significantly to the shaping of the MHC region, perhaps even as much as pathogen-driven evolution. The maintenance of pseudogene sequences, and of a certain homogeneity of the coding and non-coding sequences are both factors that would favour evolvability, and this dimension certainly adds to the complexity of MHC evolution. The overall picture we come to from the above discussion is one of balance between homogeneity and heterogeneity among the sequences of MHC molecules, with this balance being tuned towards evolvability.

## GC content as a stigma of frequent gene conversion events

As a last point of discussion, I would like to offer a potential explanation for the long standing observation that the genes coding for MHC molecules have very high GC contents, especially the sequences coding for the PBR residues of class Ia molecules [29]. According to recent reports, the direction of gene conversion is naturally biased to favour the maintenance of GC rich sequences. This bias has been proposed to counter the natural tendency of cytosine residues to mutate spontaneously into thymidine [30,31].

Biased gene conversion, which explains the high GC content of the histone and rRNA genes [30,31], may also be relevant to many other multigene families in mammals, such as insulin-like growth factors [32], polyubiquitins [33], early growth response proteins, EGR [34], protocadherins [35], Hsp70s [36] and olfactory receptors [37], as well as for certain amylase genes in Drosophila [38], and the genes of certain plant enzymes [39]. Finally, biased gene conversion towards GC has recently been proposed to play a role in the evolution of sex [40].

In the genes coding for MHC molecules, and particularly those coding for class Ia molecules, the functional new alleles resulting from events of gene conversion will be selected for by the constant pressure of pathogens, and this process could therefore account for their high GC content.

## Conclusion

*"By selecting for ever- more-devious parasites, the immune system is the cause of its own necessity." *Stephen Hedrick, 2004.

In a very nice and thought-provoking essay [41], Stephen Hedrick thus summarised his views that our immune system is far from providing an ultimate and perfect answer to the permanent challenge of infectious pathogens. Rather, he pictured the adaptive immune system as a costly and imperfect, yet indispensable, system that can never win the race against the replicative and adaptive powers of micro-organisms.

In the ancestral life forms that gave rise to all vertebrates, an adaptive immune system must, however, have conferred such a significant selective advantage that nature came up twice independently with adaptive ways to counter the capacity of infectious pathogens to evolve at striking speed. On the one hand, in the ancestor of jawless fish, an adaptive immune system arose that was based on variable lymphocyte receptors generated by somatic recombination of modules coding for leucine rich repeats [15]. On the other hand, in the ancestors to all jawed vertebrates, evolution quite rapidly gave rise to a 'canonical' adaptive immune system, complete with rag genes, T cells, B cells and an MHC region coding for both Class I and class II molecules. From what has been discussed above, we can envisage that this adaptive character of our immune system may not only have been selected to provide individuals with adaptive responses based on somatic mutations. Rather, in a further elaboration, the genomic proximity of MHC genes within the MHC region may be providing the means for a more evolvable MHC, resulting in an adaptibility of the genes that would not be immediately beneficial for individuals, but would be advantageous for species, at the level of whole populations.

## Reviewers' comments

### Reviewer's report 1

#### Stephan Beck

In their manuscript Joly and Rouillon report new evidence for the hypothesis that MHC class I genes undergo concerted evolution through gene conversion (e.g. non-homologous recombination). In support, they analysed 8 classical class I genes (termed class Ia) and 8 non-classical class I genes (termed class Ib) from primates and rodents with focus on three class Ib genes to which they refer to as CD94L family (human HLA-E, mouse H2-Qa1 and rat RT-BM1). The results are comprehensively discussed within the context of various relevant hypotheses, which adds a review-like flavour and greatly enhances the appeal of the manuscript.

Although some conclusions (and assumptions) are better supported than others, I only take issue with one particular point. Based on evidence I do not agree with, the authors assume the above mentioned CD94L family genes to represent orthologues and their many respective paralogues are not considered in subsequent analyses which may have affected some of the conclusions.

**Author response: ***Following this comment, and a suggestion made by Pierre Pontarotti on the phone, I have now modified the manuscript to remove the statement about 'the clear orthologous relationship of CD94L molecules within the primate or the rodent orders'. This is now replaced by 'There is very little room for doubt that all four primate CD94L genes descend from a common ancestral gene, and similarly for all four rodent CD94L genes'.*

On page 12, for instance, the authors conclude that at least the alpha 3 domains of the CD94L genes have all undergone intra-species concerted evolution with their respective class Ia molecules but not one of the 22 informative positions is shared across species as one would expect for orthologues. The logic conclusion would have to be that gene conversion did not occur in the ancestral (e.g. pre 80 mya) CD94L genes studied here. This is unlikely, as gene conversion has been demonstrated to be a general mechanism clearly predating the species studied here.

**Author response: ***Thanks to the process of 'open refereeing', I have been able to discuss this point with Stephan over the phone directly. His comment sprouted from some slight misunderstanding, which has now been lifted.*

The additional section (appended to main manuscript) does not really constitute a separate manuscript but adds further interesting points to the discussion and the key points could be summarized and included in the main manuscript.

**Author response: ***The solution to this has been to remove a sizeable portion of the discussion and to provide it as a clearly separate manuscript.*

The paper itself now focuses on the demonstration that HLA-E and Qa1 are orthologues. It is now much shorter, easier to read, and the message is, I hope, much clearer.

The accompanying paper is now clearly labelled as 'hypothesis', and I have used it to regroup 4 topics of discussion touching on different aspects of MHC evolution that derive from the results obtained in the paper itself, but are not directly related to these results.

### Reviewer's report 2

#### Lutz Walter

In this paper, Joly and Rouillon compare major histocompatibility complex (MHC) class I genes derived from human, non-human primates, and rodents. Based on multiple sequence alignments and phylogenetic tree reconstructions, the authors conclude that the MHC class I genes in these species are subject to concerted evolution by means of gene conversion.

One main point of criticism refers to the fact that not all known MHC class I genes of the species studied here are compared, and only a small extract from the full repertoire of class I genes was chosen for comparison. This may bias the interpretation of data. In this respect, it might be useful to concentrate on one or two species, e.g. the 'class I-rich species, mouse, rat, or rhesus monkey. In its current form, the paper contains data from a single mouse haplotype, but from several rat haplotypes. Thus, the data set should also be updated to allow the study of both inter- and intralocus gene conversion.

**Author response: ***One of the main challenges we faced when we started to do the work that would allow us to write this paper was not in terms of "How many sequences for MHC class I molecules can we collect and align ?". It was, in fact, exactly the reverse, i.e. :" With how few sequences can we proceed to demonstrate, beyond reasonable doubt, that MHC class I loci do undergo concerted evolution ?" All the figures presented in the paper were obtained from the one alignment we settled for in the end. Changing just one sequence in the list would require performing the whole study all over again, which would represent several weeks of tenuous work.*

Although our observations lead us to discuss many aspects related to MHC evolution, evaluating the frequency of inter and intra-locus conversion events was not within the remit of this study. Regarding the choice of MHC sequences from several rat MHC haplotypes, and from a single mouse haplotype, we do not see why this should have any relevance to the type of work we have done, and to the conclusions we reach. Outside of particular situation where genes can co-evolve because they are closely linked (such as RT1-A and TAP), MHC haplotypes are, after all, relatively artificial sets of genes that happened to find themselves on the same chromosomal strand when inbred strains were generated.

Starting with many more sequences, we would have faced the following problems:

1) The alignment showed on Figure 1, which was used to generate the trees, could not have been provided within the manuscript. We also find that the clarity of figures containing trees degrades rapidly when these trees have too many branches.

2) The computer time required for calculation of the trees and of the ds/dn values grows exponentially with the number of sequences, and the time spent generating the alignments and the figures is also dependent on the number of sequences included.

3) For the precise question we wanted to address, the only class Ib loci that were informative were those identified in at least two species. We also felt that it was best to restrict our analysis to those molecules for which a function had clearly been documented, and which had the same number of amino-acids as class Ia sequences (to avoid gaps in the alignment). When we embarked on this work, the only class Ib loci fulfilling these criteria were the CD94L and the murine M3 molecules. As far as we know, this is still true today.

page 16: the sister grouping of M3 and CD94L genes is due to a limited data set (see above) and does not reflect true phylogenetic relationship (and is not supported by bootstrapping). Furthermore, it contradicts data by Hurt et al. (2004) who studied the phylogenetic relationship of all rat and mouse class I genes;

**Author response: ***We are in complete agreement with the statement that the grouping of M3 and CD94L does not necessarily reflect phylogenetic relationship, and this despite a bootstrapping value of 63% (with the methods used for these comparisons, values above 60% are usually considered significant, and this is specified several times in the paper). Two alternative interpretations relating to this were (and still are) proposed in the manuscript. We actually pointed to this feature of the tree to underline our point of view that extreme caution must be exerted when carrying out phylogenetic analyses of members of multigene families that undergo extensive inter-genic exchanges.*

page 16 and more: it is not obvious why the authors introduce a new abbreviation for 'residues outside the antigen recognition site (ROARS)' and do not use the widely accepted 'non-PBR';

**Author response: ***We chose to use ROARS because we think it sounds better than 'non-PBR', and also because not all class I molecules present peptides.*

page 17, second paragraph: the authors should explain how homogenisation can be afforded in non-PBRs, particularly at those sites where PBRs and non-PBRs alternate. Is the degree of homology between the two sequences high enough to allow gene conversion to take place?

**Author response: ***What we witness here are signs that are very evocative of intra-species homogenisation, and gene conversion seems to be the most likely mechanism to explain this. We have no way of knowing when these events took place, and between what sequences (for example, some other genes, or pseudogenes, could have served as relay between certain sequences). Furthermore, although gene conversion is clearly favoured between homologous sequences, we are not aware of data documenting the minimal length of homologous sequences required for gene conversion to take place. Outside of the fact that this question seems to be way beyond the scope of our study, we therefore would have no way of addressing this question.*

I would not recommend adding of the additional section into the manuscript, as the manuscript might become 'unreadable'. However, certain aspects of this "additional" discussion section might be included in the manuscript. Nevertheless, I would strongly recommend considerable shortening of the manuscript.

In my opinion, this paper should be published, but should be regarded as a 'hypothesis paper' as it contains many assumptions, which were not proven by experimental evidence, and it contains many review-like sections.

**Author response: ***As explained above, we have managed to comply to these slightly contradictory recommendations (i.e. including more points but shortening overall) by splitting the paper in two: One 'real' paper with the results, and one hypothesis paper.*

### Reviewer's report 3

#### Pierre Pontarotti

This article hypothesizes that the Peptide Binding Region of the mouse, rat and human Class I b, that presents the leader peptide from the Class I a molecules to natural killer cells, evolved from a common ancestor while the non PBR part evolved via gene conversion.

The arguments are based upon phylogenic analysis and upon the conserved location of these MHC class I b genes.

This contrasts with another hypothesis: the MHC class I genes are lineage specific, they come from a common ancestor which is different in the human and mouse lineage, (in other word class I gene from mouse and human are paralogues), and HLA E and H2Qa1 PBR evolved via convergent evolution...

In order to strengthen their hypothesis the authors should screen other mammalian lineages using ensembl data bases since some sequences of ensembl data base are not obligatory present in NCBI NR, especially those from canis, loxodanta, bos Taurus, canis Familirais and monodelphis (even if this species is out side of the eutherian group). If an "HLAE like" PBR orthologue is found in all these groups, the author hypothesis will be stronger supported.

**Author response: ***We would indeed have been very interested to identify MHC class I molecules with CD94L-like PBR outside of the rodent and primate genera. As was already indicated in the result section entitled "Certain residues are CD94L-specific, and others are homogenised within species" (on page 10 of the current manuscript), we have repeatedly tried to identify such molecules via several approaches in all the online databases available to us, and, as of 20 Dec 2005, we have not succeeded so far.*

Second if the conversion of the non PBR HLA E like gene is an ongoing process, this could be seen at higher taxonomic level, for example at primate level by comparing human chimp and macaque MHC class I genes: more homogenization should be seen outside the "HLA E like" PBR than within the PBR.

**Author response: ***This is indeed exactly what we see, and this is discussed on page 18 of the manuscript (Comparison of this tree...... low support values).*

Other comments:

Concerning the sentence page 14 L 11: Among the classI B ...years ago. I do not understand why the results confirm that CD94L molecules are much more evolutionary conserved than Class I a molecules.

**Author response: ***This was indeed confusing, and I have tried to clarify this point by writing the following sentence:*

"The fact that the comparison of primate sequences strongly suggests that the four CD94L are orthologues, whereas this is much less clear for the corresponding class Ia sequences confirms previous reports that primate
